# HER-2 overexpressing breast cancer during pregnancy: a case report and literature review

**DOI:** 10.3389/fonc.2026.1725927

**Published:** 2026-01-29

**Authors:** Si Li, Tuoshu Huang, Mujin Feng, Minjun Deng, Xiyu Chen, Xinning Li, Dan Mo

**Affiliations:** Department of Breast Surgery, Maternal and Child Health Hospital of Guangxi Zhuang Autonomous Region, Nanning, China

**Keywords:** breast cancer during pregnancy, case report, fetal safety, HER-2 overexpressing, neoadjuvant chemotherapy in pregnancy

## Abstract

**Background:**

Breast cancer during pregnancy (PrBC) is rare but increasingly reported due to delayed childbearing, widespread assisted reproduction, and younger onset of breast cancer. Among these, HER2-overexpressing subtypes pose particular clinical challenges in balancing effective oncologic control with fetal safety. It requires a delicate balance between optimizing maternal oncologic outcomes and ensuring fetal safety.

**Case presentation:**

We report the case of a 33-year-old woman diagnosed with HER-2 overexpressing invasive ductal carcinoma of the right breast at 16 weeks of gestation. Driven by a strong desire to continue the pregnancy, the patient, in consultation with a multidisciplinary team, opted for neoadjuvant chemotherapy. From 17 to 31 weeks’ gestation, she received four cycles of epirubicin and cyclophosphamide, followed by one cycle of nab-paclitaxel, achieving a partial response. At 37 weeks, she underwent a successful vaginal delivery, giving birth to a healthy female infant. Postpartum, she continued her neoadjuvant treatment with three cycles of nab-paclitaxel plus dual anti-HER2 therapy (trastuzumab and pertuzumab). After completing the full neoadjuvant regimen, she underwent breast-conserving surgery, and pathology confirmed a complete response. Her postoperative treatment included adjuvant dual anti-HER2 therapy and whole-breast radiotherapy. At the last follow-up (18 months post-delivery), the mother showed no signs of recurrence, and the child exhibited normal growth and neurodevelopment.

**Conclusions:**

This case demonstrates that with careful multidisciplinary planning and individualized treatment strategies, it is feasible to achieve both successful maternal oncologic control and the delivery of a healthy baby in patients with HER-2 overexpressing breast cancer during pregnancy. This case contributes valuable evidence to the management of this complex clinical scenario.

## Introduction

Breast cancer is one of the most common malignant tumors during pregnancy, but the incidence of breast cancer during pregnancy is approximately 1/3000, with a median age at diagnosis of 32 years (1). Breast cancer diagnosed during pregnancy accounts for 0.2%–3.8% of all breast cancer patients and about 4% of breast cancer patients under 45 years of age (2, 3). With the postponement of childbearing age, the widespread use of assisted reproductive technologies, and the trend toward younger cancer patients, the prevalence of breast cancer diagnosed during pregnancy (PrBC) is gradually increasing.

The clinical management of PrBC is highly complex, as it requires simultaneous consideration of maternal prognosis and fetal safety. Furthermore, most PrBC cases are diagnosed at an advanced stage, which further exacerbates the therapeutic challenge (4, 5). Here, we present the case of a woman with a strong desire to continue her pregnancy, who was unexpectedly diagnosed with HER2-overexpressing breast cancer with ipsilateral axillary lymph node metastasis in the second trimester. This report aims to highlight the importance of individualized treatment strategies for PrBC to optimize both maternal and fetal outcomes (6).

## Case presentation

A 33−year−old woman (G3P1) presented at 16 weeks’ gestation with a painless right breast mass. Notably, she had one prior live birth and a medical termination of pregnancy in November 2022 due to a diagnosis of fetal trisomy 18. There was no personal or family history of malignancy.

**Figure 1:**
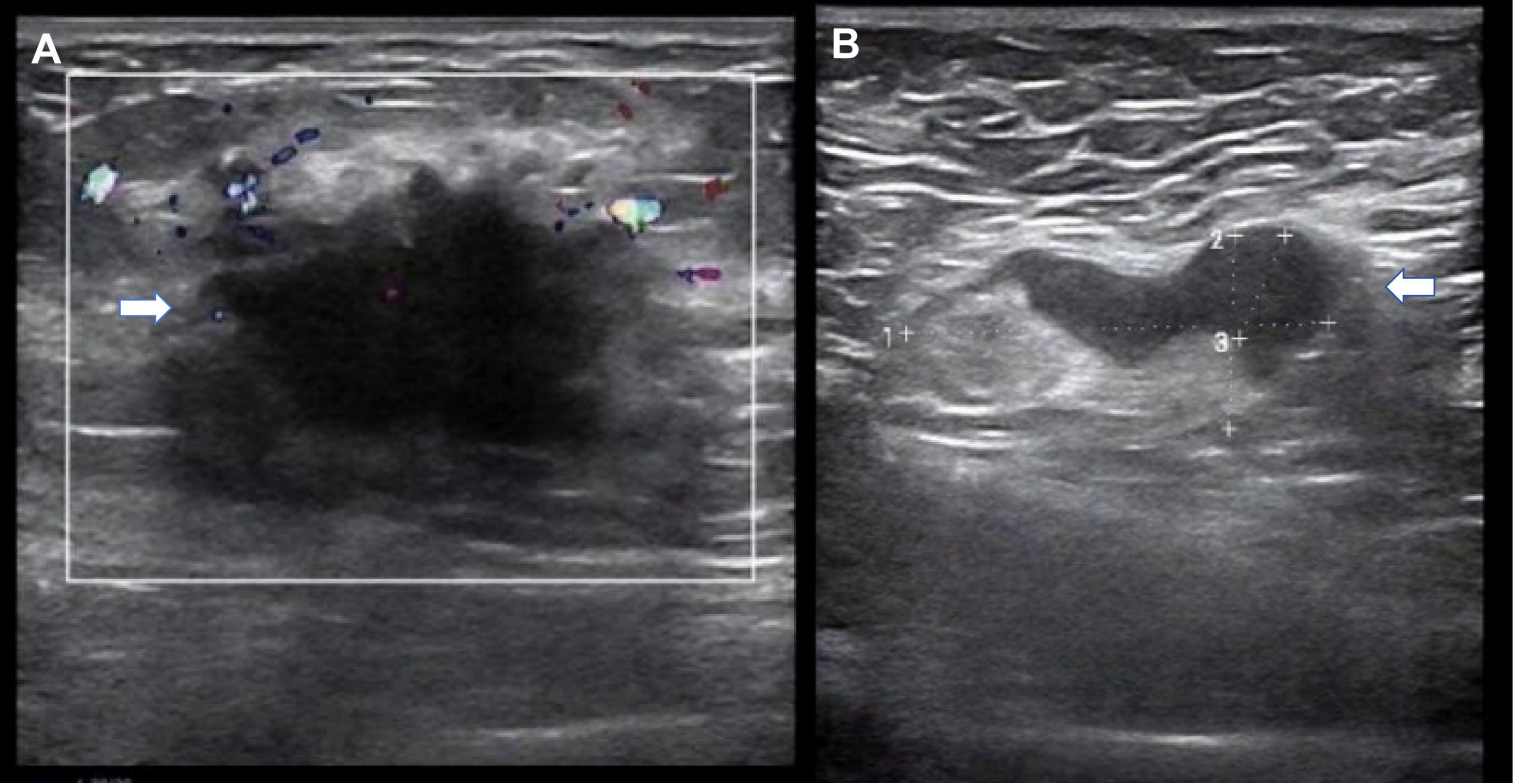
Ultrasound images of the right breast and axilla at diagnosis (16 weeks of gestation). **(A)** The arrow indicates a hypoechoic, irregular mass located in the upper outer quadrant of the right breast, measuring 3.2×2.1 cm. **(B)** The arrow points to an enlarged right axillary lymph node measuring 3.4 cm in maximum diameter, showing cortical thickening suggestive of metastasis.

On physical examination, a 3.0 × 3.0 cm firm ill-defined mass was palpated in the upper outer quadrant of the right breast, along with a 3.0 cm mobile right axillary mass. Breast ultrasound showed a hypoechoic lesion at 9–10 o’clock measuring 3.2 × 2.1 cm (**Figure 1**) and multiple enlarged right axillary lymph nodes (the largest measuring 3.4 cm). Obstetric ultrasound confirmed a singleton fetus consistent with 16 weeks’ gestation. Cervical and abdominal ultrasonography revealed no evidence of metastatic disease. Core needle biopsies of the right breast mass and right axillary lymph node were performed on October 7, 2023. Pathological examination indicated invasive carcinoma of no special type (grade 2) in the right breast, without vascular or nerve invasion. Cancer metastasis was detected in the right axillary lymph node. Immunohistochemistry(IHC) results showed negative estrogen receptor (ER), negative progesterone receptor (PR), and over-expression of human epidermal growth factor receptor 2 (HER-2).

**Figure 2:**
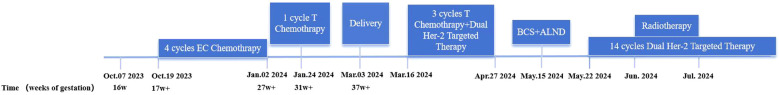
Treatment course timeline. EC, epirubicin and cyclophosphamide; T, taxane (nab-paclitaxel); BCS, breast-conserving surgery; ALND, axillary lymph node dissection. Dual HER-2 targeted therapy refers to the combination of trastuzumab and pertuzumab.

Following extensive counseling, the patient expressed a strong desire to continue the pregnancy, a decision deeply influenced by her previous pregnancy loss. After a multidisciplinary team (MDT) discussion, a collaborative decision was made to honor the patient’s wish and proceed with a treatment plan compatible with the ongoing pregnancy. The treatment timeline is summarized in **Figure 2**. Between 17 and 31 weeks of gestation, the patient underwent four cycles of epirubicin–cyclophosphamide (epirubicin 100 mg/m²+ cyclophosphamide 600 mg/m²) followed by one cycle of nab−paclitaxel (260 mg/m²), each administered at standard doses on a 21−day schedule. Tumor response during pregnancy was assessed via breast and axillary ultrasonography at each cycle; the imaging demonstrated a progressive reduction in lesion size, consistent with a partial response per RECIST criteria. The treatment was well-tolerated overall, with adverse events limited to grade 1–2 myelosuppression and mild gastrointestinal symptoms (**Table 1**). During chemotherapy, the patient also received regular psychological counseling and support, and obstetric ultrasound was performed every 3–4 weeks to assess the fetal status. We did not use non-invasive prenatal testing to assess whether the fetus was developing normally. The fetal biparietal diameter, head circumference, femur length, and amniotic fluid volume were all within the normal range, suggesting no evidence of fetal growth restriction. Furthermore, throughout the treatment course, no unanticipated adverse events were observed, such as severe maternal infection or acute psychological crisis. At 37 weeks of gestation, the patient delivered a healthy female infant (birth weight: 2730 g) via spontaneous vaginal delivery.

**Table 1:** Treatment-related adverse events during neoadjuvant chemotherapy in pregnancy (graded according to CTCAE v5.0).

System organ class/Adverse event	CTCAE grade	Management & outcome
Blood and lymphatic system disorders		
Myelosuppression (Leukopenia/Neutropenia)	1-2	Managed with short-term G-CSF support.
Anemia	1	Observation.
Gastrointestinal disorders		
Nausea	1	Controlled with antiemetics (Ondansetron).
Vomiting	1	Controlled with antiemetics.
Musculoskeletal and connective tissue disorders		
Myalgia/Bone pain	1	Supportive care; Spontaneous resolution.
Skin and subcutaneous tissue disorders		
Alopecia	2	Patient counseled.

CTCAE, Common Terminology Criteria for Adverse Events (Version 5.0). Data represent the highest grade observed during the treatment course. G-CSF: Granulocyte colony-stimulating factor.

Postpartum contrast−enhanced CT revealed no distant metastases. On postpartum day 11, she began a 21−day neoadjuvant cycle consisting of targeted therapy on day 1 (trastuzumab: loading dose 8 mg/kg, maintenance dose 6 mg/kg; pertuzumab: loading dose 840 mg, maintenance dose 420 mg) followed on day 2 by nab−paclitaxel at 260 mg/m². Three such cycles were administered. Between delivery and breast surgery, treatment response was monitored primarily via breast MRI; the imaging showed a progressive reduction in both the primary breast lesion and axillary lymph nodes, consistent with a partial response per RECIST criteria. She subsequently underwent right breast lumpectomy with axillary lymph node dissection. Final pathology revealed no residual invasive or *in situ* carcinoma (ypT0N0), confirming pathological complete response(pCR).

**Figure 3:**
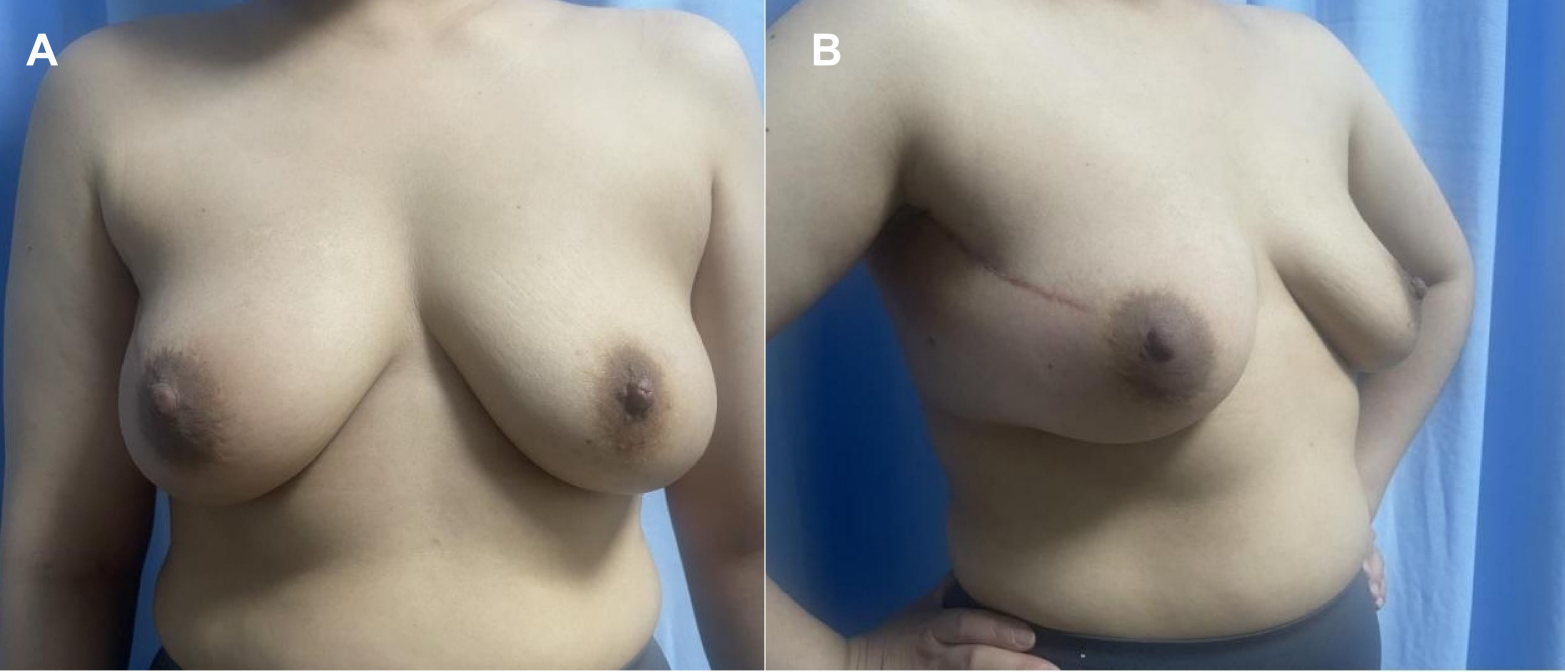
Postoperative appearance of the right breast 5 months after breast- conserving surgery. **(A)** Anterior view. **(B)** Lateral view showing the surgical incision.

She completed adjuvant whole−breast radiotherapy and the planned course of dual anti−HER2 therapy (trastuzumab plus pertuzumab). The right breast appearance at 5 months after surgery is shown in **Figure 3**. Follow-up of the child up to 18 months of age has shown normal physical growth and development, with the Gesell Developmental Schedules indicating age-appropriate intellectual and emotional development. From the onset of the disease to the last follow-up on October 1, 2025, the patient has shown no obvious signs of tumor recurrence, and the disease-free survival (DFS) is ongoing. During the 18-month follow-up, the patient maintained regular contact with the medical team, frequently sharing photographs of her daughter’s growth and expressing gratitude for the successful preservation of her pregnancy. This case demonstrates that a carefully tailored multidisciplinary plan can address the complex clinical needs of breast cancer diagnosed during pregnancy, achieving a rare combination of favorable outcomes: term delivery of a healthy infant, complete eradication of detectable disease, and successful breast conservation.

## Discussion

Breast cancer diagnosed during pregnancy (PrBC) should be clearly distinguished from postpartum breast cancer owing to their differences in biological behavior and clinical priorities (7–9). Traditionally, given the similar endocrine environment, pregnancy-associated breast cancer (PABC) was defined as breast cancer diagnosed during pregnancy or within one year postpartum (10). As highlighted in a large international cohort study, prior research often pooled PrBC and postpartum breast cancer under the definition of PABC, obscuring distinct prognostic patterns. Notably, Amant et al. showed that adverse outcomes predominantly reflect the postpartum subset, whereas prognosis for PrBC per se is not significantly worse than for age-matched controls (11). Increasingly, research supports abandoning the umbrella definition in favor of separately classifying PrBC and postpartum breast cancer, as these conditions have divergent biological features, prognoses, and therapeutic considerations—particularly regarding pharmacologic interventions.

During pregnancy, women typically experience physiological changes such as breast hypertrophy, increased glandular density, and changes in the nipples. These physiological changes in the breast during pregnancy combined with limitations on diagnostic imaging increase the difficulty of detecting breast cancer. Consequently, breast cancer diagnosed during pregnancy is often diagnosed at a locally advanced stage, characterized by larger tumor size or lymph node involvement (3). Concurrently, the critical need to balance effective tumor control with maternal and fetal safety profoundly complicates clinical decision-making, rendering PrBC a highly challenging clinical issue where optimal diagnostic and therapeutic strategies remain a subject of ongoing research (12).

In treatment decision-making, first and foremost, it is critical to ascertain the patient’s desire regarding pregnancy continuation. Theoretically, there is no conclusive evidence indicating that terminating pregnancy can improve the patient’s survival (13, 14). As reported in previous studies, some scholars have suggested that, in selected cases of highly aggressive malignancies, termination of pregnancy should be considered to allow timely administration of uncompromised treatment and to avoid missing a potential window for cure. However, when pregnant patients are diagnosed with highly aggressive breast cancer or non-early stage cancer, careful consideration must be given to whether to continue the pregnancy. For instance, the HER-2 overexpressing patients in question require anti-HER-2 targeted therapies that are contraindicated during pregnancy (15–17). If the pregnancy is to be continued, it is essential to ensure that non-standard treatment regimens that do not include anti-HER-2 targeted therapy can still achieve effective tumor control. The treatment of breast cancer during pregnancy should be tailored to develop individualized regimens based on the patient’s clinical stage, tumor biological characteristics, and gestational age (4). It emphasizes personalized management through a multidisciplinary team collaboration model, with full respect for the patient’s preferences (18).

The key to the correct management in this case lies in balancing oncologic efficacy with fetal safety, particularly in the context of a second-trimester pregnancy and Her-2 overexpressing breast cancer with axillary lymph node metastasis. Guidelines and consensus delineate several potential treatment pathways, each with major trade-offs. First, termination of pregnancy permits the use of standard-of-care systemic and targeted therapies, including trastuzumab and pertuzumab, thereby potentially improving maternal malignant disease control. However, choosing abortion directly opposes the patient’s strong desire to maintain the pregnancy and may exact significant psychological burden. Second, proceeding immediately with radical surgery during pregnancy is technically feasible and relatively safe in the second trimester. Owing to the urgency of the patient’s tumor and pregnancy, her surgical approach is limited to mastectomy and axillary lymph node dissection. These operations would eliminate breast-conserving options and carry risks including lymphedema, sensory deficits, and impaired upper limb function. Third, initiating anti-HER2 targeted therapy (e.g., trastuzumab, pertuzumab) during pregnancy—which could yield maximal tumor response—is strictly contraindicated by all major guidelines due to well-documented fetal toxicity, namely oligohydramnios, renal dysgenesis, and neonatal complications (15).

In the case of our patient, the treatment strategy not only aligns with evidence-based principles for managing gestational breast cancer but also accommodates the patient’s personalized needs stemming from her strong desire to continue the pregnancy. Clinical data demonstrate that anthracycline- and taxane-based chemotherapy is safe when administered during the second trimester, with no significant increase in fetal anomalies or adverse pregnancy outcomes (19–21). A notable modification in our regimen was the use of nab-paclitaxel. We acknowledge that current major guidelines generally recommend weekly solvent- based paclitaxel as the preferred taxane formulation during pregnancy due to its well-established safety profile. However, the choice of nab-paclitaxel in this case was individualized. This decision was primarily based on the solvent-free nature of nab-paclitaxel, which eliminates the risk of hypersensitivity reactions and, crucially, removes the requirement for high-dose corticosteroid premedication. Minimizing steroid exposure was a priority to reduce the risk of gestational diabetes and maternal immunosuppression in our patient. Although data on nab-paclitaxel in pregnancy are less robust than for solvent-based paclitaxel, preliminary reports indicate limited placental transfer due to its high molecular weight and protein binding (22). Recent studies continue to support the safety of taxanes in the second and third trimesters, showing no significant increase in congenital malformations or adverse obstetric outcomes compared to standard regimens (23, 24). In our case, the treatment was well- tolerated with no adverse fetal sequelae, though we emphasize that nab-paclitaxel remains an investigational alternative in pregnancy and warrants cautious use with close fetal monitoring. Therefore, a tailored neoadjuvant chemotherapy regimen was selected to balance systemic tumor control with the healthy development of the fetus during pregnancy. Following delivery, dual-targeted therapy was administered to further enhance tumor control. Additionally, neoadjuvant therapy provides a superior opportunity to optimize surgical strategies. This approach achieves the critical balance between controlling malignancy and allowing the pregnancy to progress to a viable gestational age, optimizing outcomes for both the mother and the fetus.

The choice of chemotherapy regimen is also a key consideration. While our patient tolerated the EC regimen well, it is important to consider alternatives for patients who may experience contraindications like cardiotoxicity or severe adverse reactions. For HER-2 positive hormone receptor negative patients in a non-pregnant setting, a TCbH-P (Docetaxel, Carboplatin, Trastuzumab, Pertuzumab) neoadjuvant regimen is common (19, 25, 26). In pregnancy, the use of carboplatin remains controversial (4). In situations where anthracyclines are contraindicated—such as documented hypersensitivity or pre-existing cardiac dysfunction—single-agent taxane chemotherapy is generally considered the preferred option (23), given its established safety profile. Weekly paclitaxel, in particular, is often better tolerated and does not require steroid premedication. However, in patients with a high tumor burden and aggressive molecular subtypes, especially triple-negative breast cancer, a modified TCb regimen (docetaxel plus carboplatin) may be a potential alternative. While some reports, particularly in ovarian cancer, have demonstrated reassuring short-term fetal outcomes with carboplatin (1, 27), it is crucial to acknowledge that preclinical data indicate significant transplacental transfer (28). Furthermore, a documented case of cisplatin-associated fetal ototoxicity serves as a cautionary tale (29). A prudent strategy, therefore, might involve short-term carboplatin use in selected high-risk subtypes—such as triple-negative disease or following four cycles of anthracyclines—only after a thorough risk–benefit discussion with the patient.

Regarding chemotherapy, we administered a standard dose of epirubicin and cyclophosphamide. This decision was based on established pharmacokinetic principles in pregnancy. Pregnant patients exhibit a larger volume of distribution and increased placental metabolism, which can lead to lower peak plasma concentrations and faster clearance of chemotherapeutic agents compared to non-pregnant individuals (28, 30). Dose reduction is generally not recommended as it may compromise maternal outcomes without providing a significant fetal safety benefit; nevertheless dose adjustments may be necessary for toxicity management or altered organ function. Supportive care during chemotherapy was pivotal for ensuring patient safety and treatment adherence. Her chemotherapy-induced nausea and vomiting (CINV) were effectively managed with ondansetron and methylprednisolone (31), both of which are considered safe during pregnancy. She also experienced grade 1–2 myelosuppression, which was managed with short-term granulocyte colony-stimulating factor (G-CSF) support. Evidence supports that G-CSF can be safely administered during pregnancy if clinically indicated to manage severe neutropenia (32), as its transplacental passage is minimal and does not appear to pose a considerable fetal risk.

A critical aspect of management is fetal monitoring. In our patient, fetal well-being was assessed exclusively through serial obstetric ultrasounds, which consistently showed normal growth and development. We deliberately avoided non-invasive prenatal testing (NIPT). While NIPT is a standard method for detecting fetal aneuploidies by analyzing cell-free DNA (cfDNA) in maternal blood, its utility in pregnant cancer patients is questionable. The presence of circulating tumor DNA (ctDNA) from the mother’s cancer can confound the results, leading to a high rate of false positives that might incorrectly suggest a fetal genetic abnormality rather than reflecting the maternal disease (33, 34). Therefore, informing patients about this limitation is essential, and reliance on morphological assessment via ultrasound remains the preferred strategy (4).

Beyond these clinical protocols, we must address the profound psychological burden carried by the patient. As we observed in our case, integrating psychological counseling from the moment of diagnosis is a fundamental component of care (35). These patients often face a triad of immense pressures: the internal anxiety of their own cancer diagnosis, the external judgment or lack of understanding from others for choosing to continue the pregnancy, and a heavy sense of guilt and worry about the potential effects of chemotherapy on their unborn child (36). Navigating this is not merely a matter of emotional stress; it is a profound challenge to their identity as a patient, a mother, and a person. The successful outcome in our case—a healthy mother on the path to recovery and a thriving child—is therefore not only a medical achievement but also a testament to the patient’s resilience, fortified by an integrated, multidisciplinary support system that addressed both her physical and deep-seated psychological needs. That said, it is crucial to acknowledge the inherent limitations of this single case report, which restricts the generalizability of our findings. The successful tumor response to neoadjuvant therapy observed in this particular patient, while encouraging, may not be universally replicated; indeed, cases of disease progression or insufficient response during treatment can occur and demand meticulous clinical observation and rapid adjustment of highly individualized, tailored treatment plans. These observations underscore the critical need for larger, prospective studies to validate optimal multidisciplinary management strategies and improve outcomes for this vulnerable patient population.

## Conclusion

Breast cancer diagnosed during pregnancy represents a highly complex and challenging clinical scenario. An optimal treatment strategy should be guided by multiple factors, including the patient’s preferences (continuation of pregnancy and breast appearance), tumor profile (tumor stage and subtype), and gestational age. Successful management requires close collaboration among a multidisciplinary team and the implementation of individualized treatment plans. Such an approach aims to maximize maternal survival and quality of life while ensuring the optimal health and development of the fetus.

## Data Availability

The original contributions presented in the study are included in the article/Supplementary Material. Further inquiries can be directed to the corresponding author.
